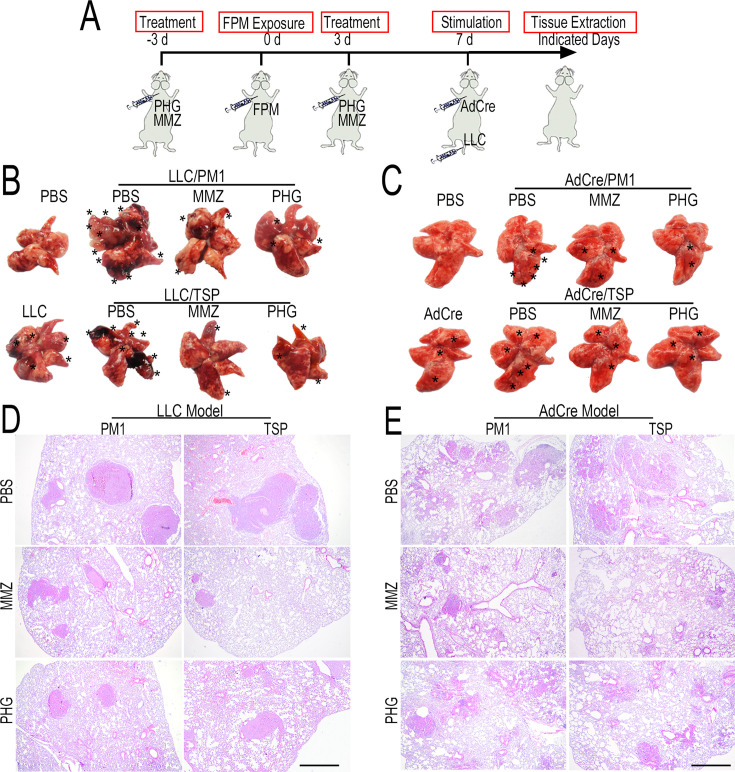# Correction: Air pollution particles hijack peroxidasin to disrupt immunosurveillance and promote lung cancer

**DOI:** 10.7554/eLife.108912

**Published:** 2025-08-20

**Authors:** Zhenzhen Wang, Ziyu Zhai, Chunyu Chen, Xuejiao Tian, Zhen Xing, Panfei Xing, Yushun Yang, Junfeng Zhang, Chunming Wang, Lei Dong

**Keywords:** Mouse

Wang Z, Zhai Z, Chen C, Tian X, Xing Z, Xing P, Yang Y, Zhang J, Wang C, Dong L. 2022. Air pollution particles hijack peroxidasin to disrupt immunosurveillance and promote lung cancer. *eLife* **11**:e75345. doi: 10.7554/eLife.75345.

Published 19 April 2022

After publication we discovered we inadvertently used incorrect overlapping images representing different experimental conditions. Specifically, errors were introduced into some of fluorescence staining images in Figure 1E, Figure 3B and in Figure 1—figure supplement 6; transmission electron microscopy images in Figure 1—figure supplement 1B; the Masson’s trichrome histological images in Figure 5—figure supplement 3A; and hematoxylin and eosin (H&E) staining images in Figure 5—figure supplement 8D and E.

Upon careful reanalysis of these images, we found the errors arose during figure preparation. We captured multiple fields of views from the same samples. Our use of abbreviated labelling meant that images had similar file names, which resulted in inadvertent mix-ups where overlapping images were erroneously introduced to the wrong figures. These issues resulted from organizational oversights in handling a large volume of image data. We sincerely apologize for any confusion these errors may have caused and for not identifying them prior to publication. We have now corrected these figures and related analysis. Importantly, these corrections do not affect our conclusions. No changes have been made to the main text or figure legends of the paper.

**Correction #1:** To address overlap between EdU stained panels (PBS and TSP) in Figure 1E.

The corrected Figure 1 with updated panel E is shown here:

**Figure 1:**
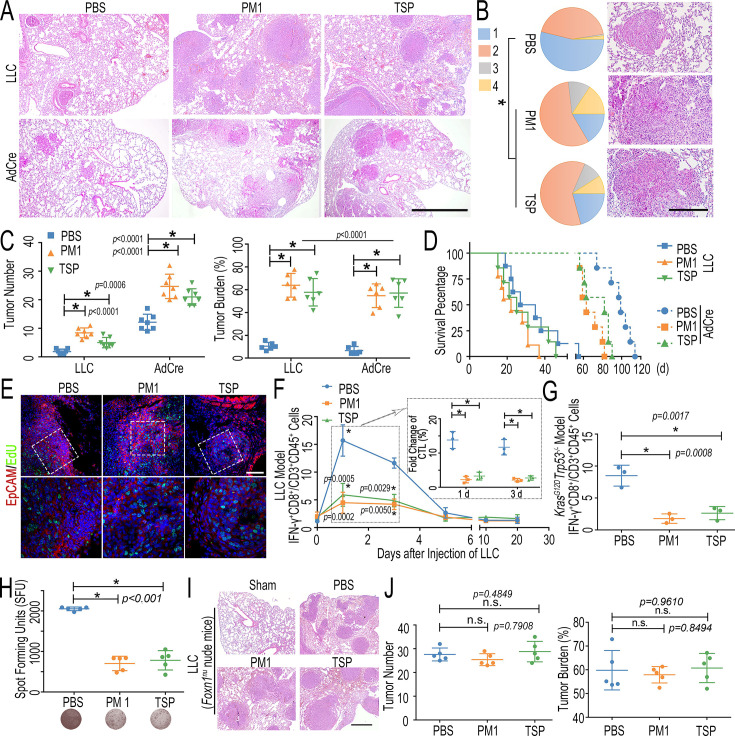


The originally published Figure 1 is shown for reference:

**Figure 2:**
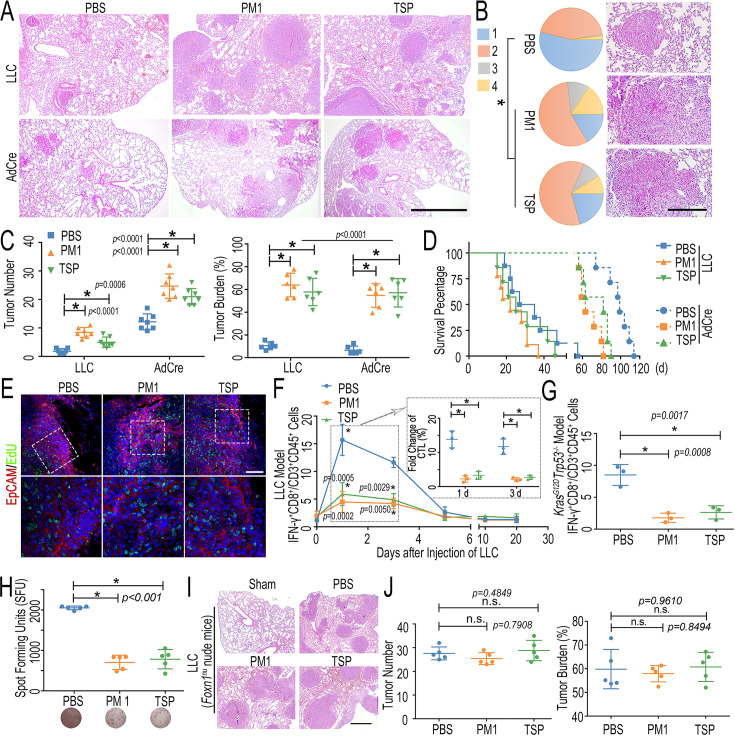


**Correction #2:** To address overlap between LH-FPM groups (PM1 and TSP) in Figure 3B and correct the related analysis in Figure 3C–E.

The corrected Figure 3 with updated panel B, C, D and E is shown here:

**Figure 3:**
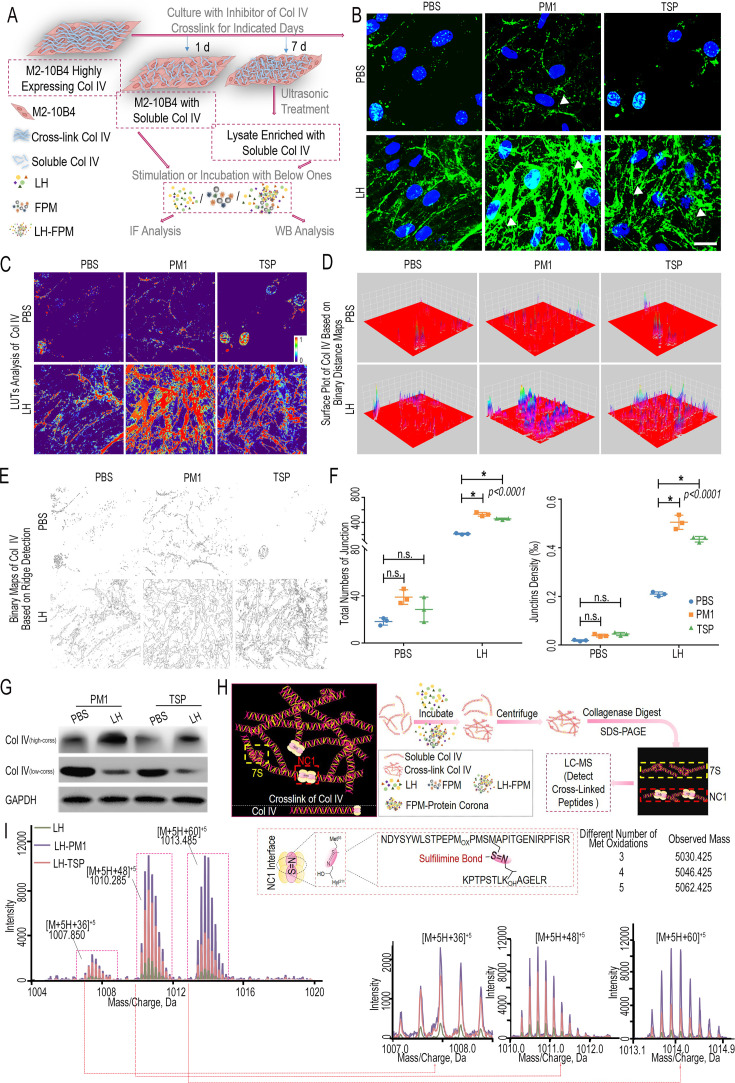


The originally published Figure 3 is shown for reference:

**Figure 4:**
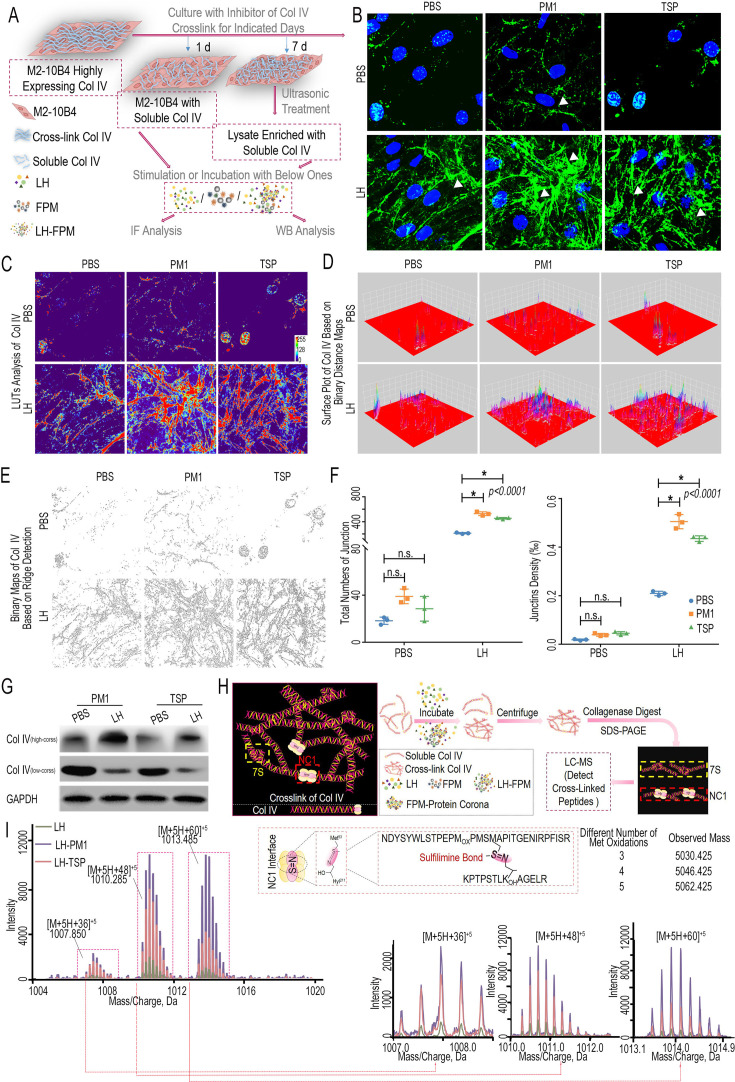


**Correction #3:** To address overlap (between Gachun (GC) and Gulou (GL), as well as Suzhou (SZ) and Pulou (PK)) in Figure 1—figure supplement 1B.

The corrected Figure 1—figure supplement 1 with updated panel B is shown here:

**Figure 5:**
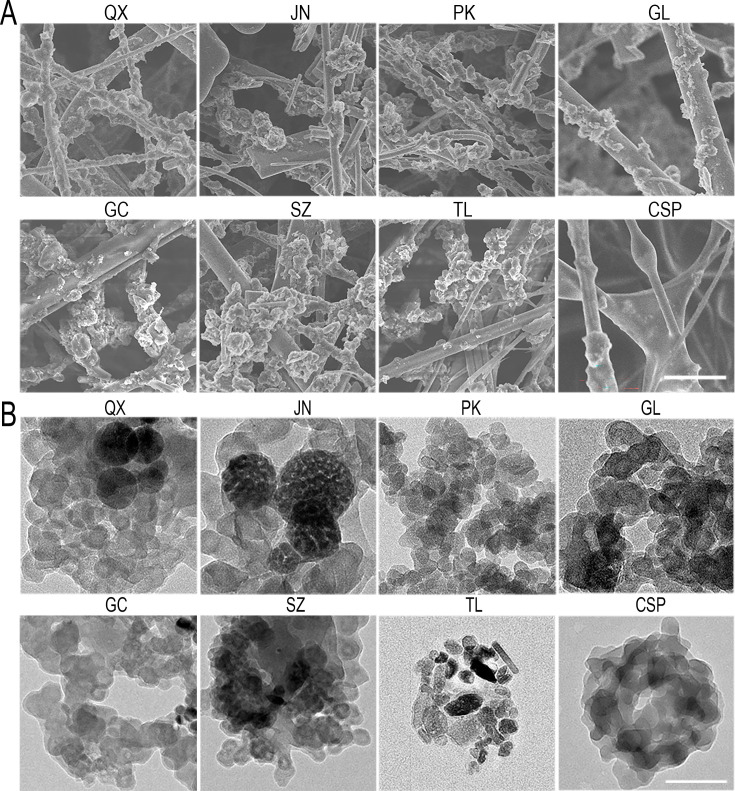


The originally published Figure 1—figure supplement 1 is shown for reference:

**Figure 6:**
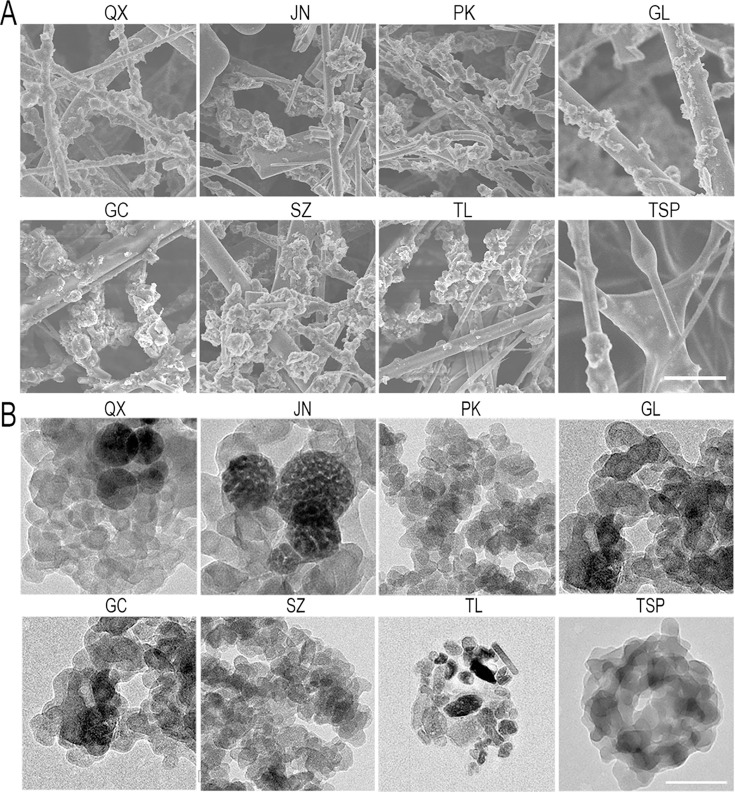


**Correction #4:** To address overlap between TSP (10 days) and PM1 (20 days) in Figure 1—figure supplement 6.

The corrected Figure 1—figure supplement 6 is shown here:

**Figure 7:**
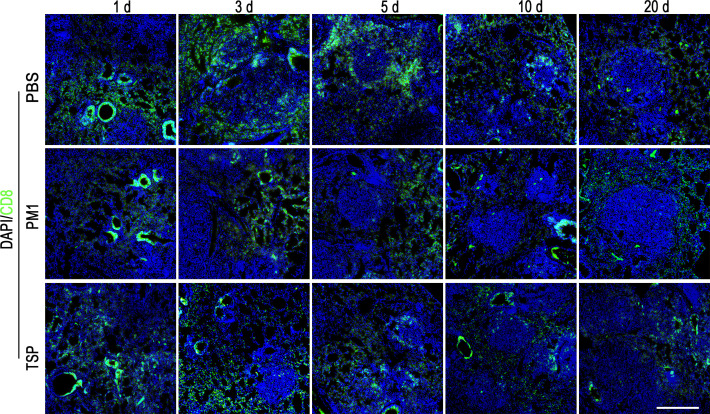


The originally published Figure 1—figure supplement 6 is shown for reference:

**Figure 8:**
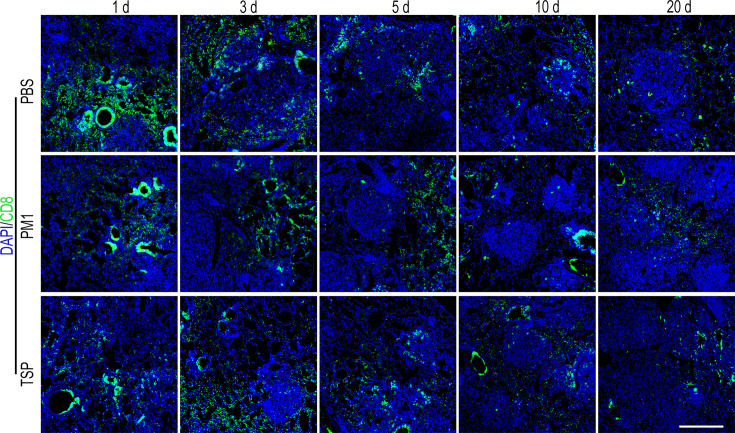


**Correction #5:** To address overlap between PM1 and TSP group administered with shNC in Figure 5—figure supplement 3A.

The corrected Figure 5—figure supplement 3 with updated panel A is shown here:

**Figure 9:**
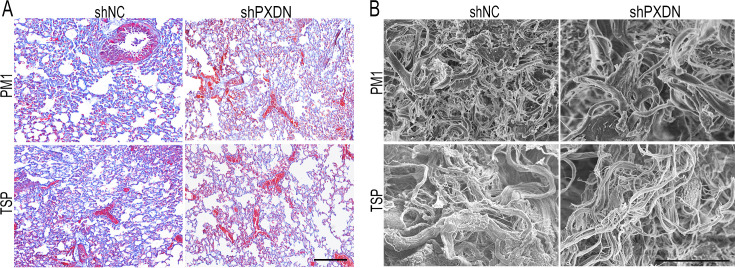


The originally published Figure 5—figure supplement 3 is shown for reference:

**Figure 10:**
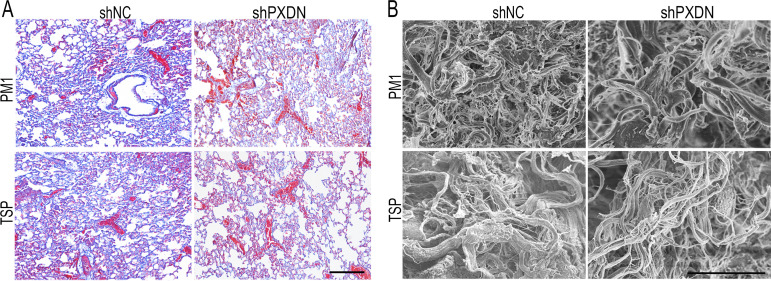


**Correction #6:** To address overlap between Figure 5—figure supplement 8D (LLC model / PM1 group administered with MMZ) with Figure 1B (AdCre model exposed to PBS). Also, overlap between Figure 5—figure supplement 8E (AdCre model with TSP exposure treated with PBS) with Figure 1A (AdCre model with TSP exposure) and AdCre model / PM1 group administered with MMZ with Figure 5E (LLC model with TSP exposure administered with shPXDN).

The corrected Figure 5—figure supplement 8 with updated panels D and E is shown here:

**Figure 11:**
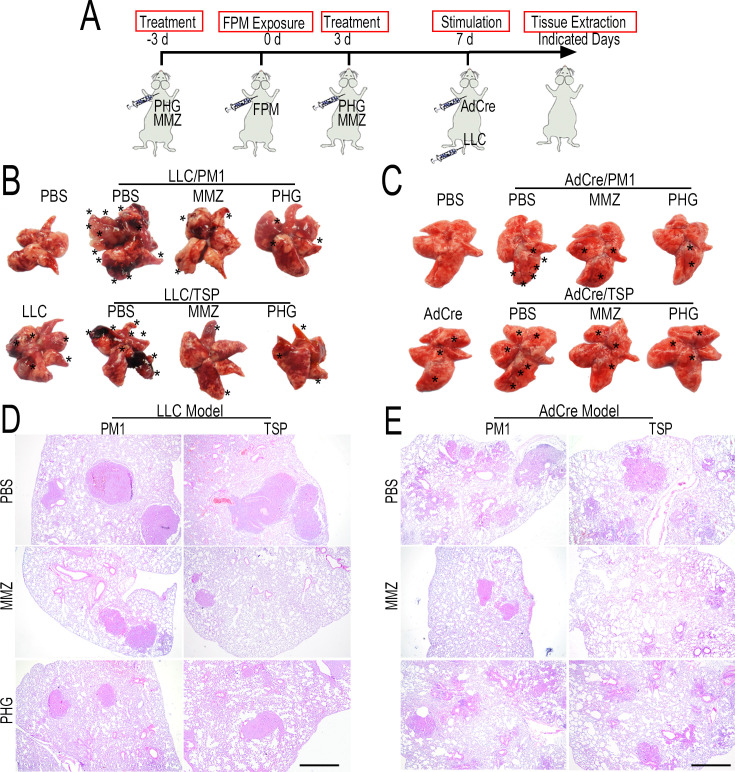


The originally published Figure 5—figure supplement 8 is shown for reference:

**Figure 12:**