# Supplementary figures and images for: Air pollution particles hijack peroxidasin to disrupt immunosurveillance and promote lung cancer
Source: eLife. 2022 Apr 19;11:e75345. doi: 10.7554/eLife.75345 (PMC9054135; doi:10.7554/eLife.75345)

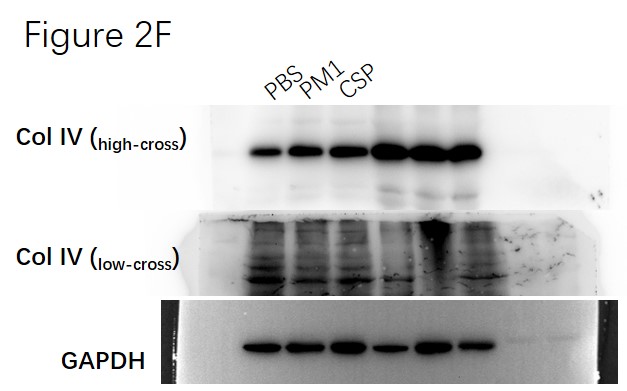

Supplement: Source data 1. [file elife-75345-data1.zip › source data of gels and blots/Figure 2F.jpg]

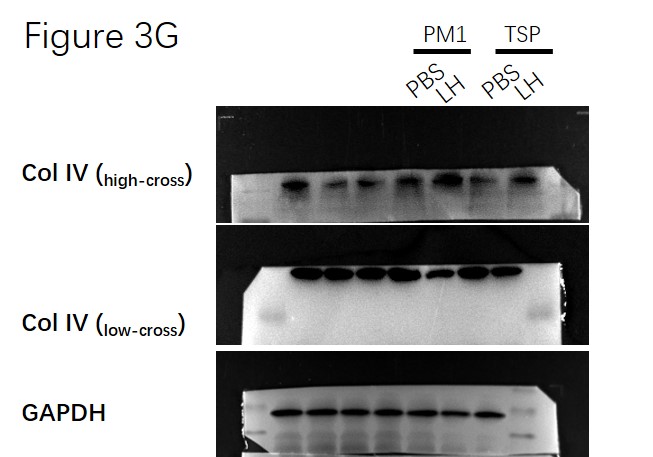

Supplement: Source data 1. [file elife-75345-data1.zip › source data of gels and blots/Figure 3G.jpg]

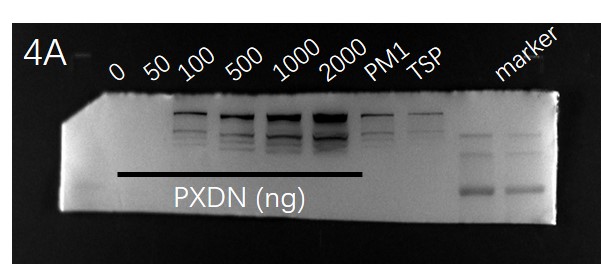

Supplement: Source data 1. [file elife-75345-data1.zip › source data of gels and blots/Figure 4A.jpg]

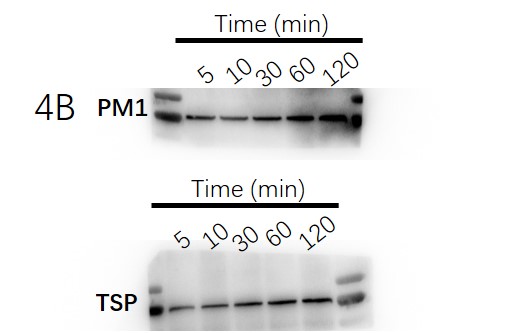

Supplement: Source data 1. [file elife-75345-data1.zip › source data of gels and blots/Figure 4B.jpg]

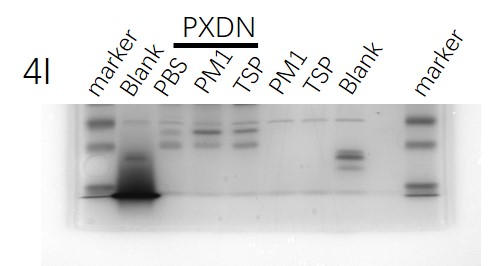

Supplement: Source data 1. [file elife-75345-data1.zip › source data of gels and blots/Figure 4I.jpg]
